# Contrast‐Enhanced Ultrasound in Diagnosing Rare Calf Muscle Metastasis of Lung Cancer: A Case Report With Literature Review

**DOI:** 10.1002/ccr3.71040

**Published:** 2025-09-26

**Authors:** Yiqun Lin, Shuai Zheng, Hongxia Zhang, Sen Wang, Wen He, Wei Zhang

**Affiliations:** ^1^ Department of Ultrasound Beijing Tiantan Hospital, Capital Medical University Beijing China

**Keywords:** calf swelling, contrast‐enhanced ultrasound, lung cancer, skeletal muscle metastasis

## Abstract

Skeletal muscle metastasis, though rare in lung cancer, can mimic thromboembolic disease. We describe a patient with squamous cell carcinoma who developed bilateral calf swelling postoperatively. While ultrasound revealed right popliteal vein thrombosis, a left calf hypoechoic lesion exhibited marked hypervascularity on contrast‐enhanced and microvascular flow imaging, ultimately diagnosed as metastasis. This case illustrates a critical diagnostic pitfall and advocates for contrast‐enhanced ultrasound in evaluating ambiguous limb lesions during cancer care.

**Abbreviations**
CDFI: color Doppler Flow Imaging
CEUS: contrast‐enhanced ultrasound
DVT: deep vein thrombosis
LC: lung cancer
SMI: superb microvascular imaging
SMM: skeletal muscle metastasis

**Summary**

- This instructive case demonstrates the diagnostic efficacy of contrast‐enhanced ultrasound in detecting rare calf muscle metastasis from lung cancer.
- Clinicians should consider metastatic disease in patients with unexplained muscle masses and a history of malignancy.
- Contrast‐enhanced ultrasound provides a valuable complementary imaging modality, enhancing early detection of atypical metastases.

## 1 Background

Lung cancer (LC) demonstrates remarkable metastatic potential, though skeletal muscle metastasis (SMM) represents a rare manifestation, comprising only 0.1%–2% of metastatic cases. This apparent rarity contrasts sharply with autopsy findings revealing a 16%–17.5% prevalence, a discrepancy likely attributable to SMM's frequently nonspecific clinical and radiographic presentation [1]. The diagnostic challenge is particularly evident when evaluating cancer patients presenting with only limb swelling, where deep vein thrombosis (DVT) typically dominates clinical consideration. Our case provides crucial counterpoint evidence that SMM may underlie such presentations, while simultaneously demonstrating the diagnostic utility of contrast‐enhanced ultrasound (CEUS) in identifying these atypical metastatic lesions.

## 2 Case Presentation

**FIGURE 1:**
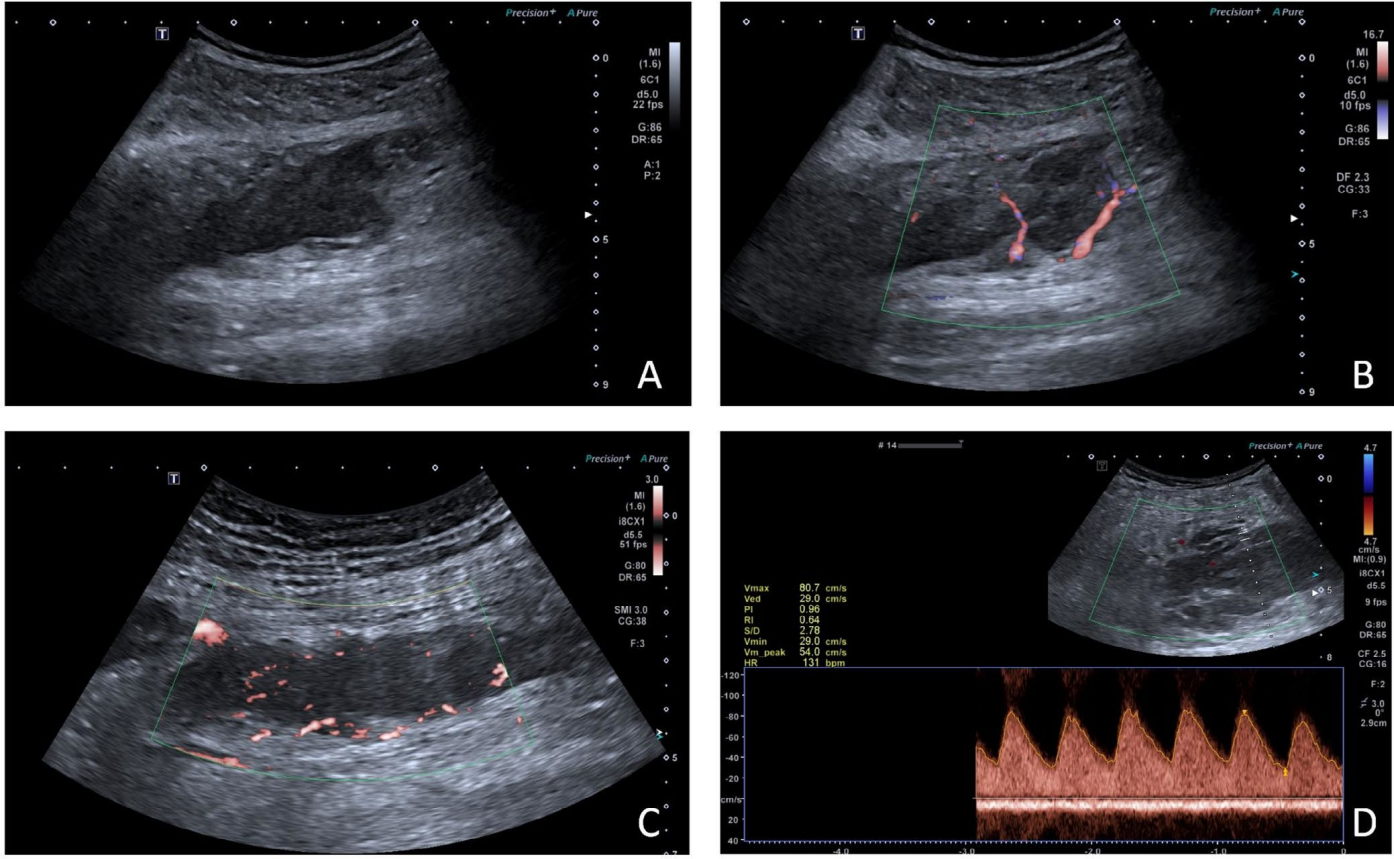
(A) Hypoechoic area within the calf muscles, with unclear borders and irregular shape. (B) CDFI shows strip‐like blood flow signals within the lesion. (C) SMI demonstrates blood flow signals within the lesion; (D) Pulsed Wave Doppler (PW) shows a high‐speed, low‐resistance blood flow spectrum within the lesion.

**FIGURE 2:**
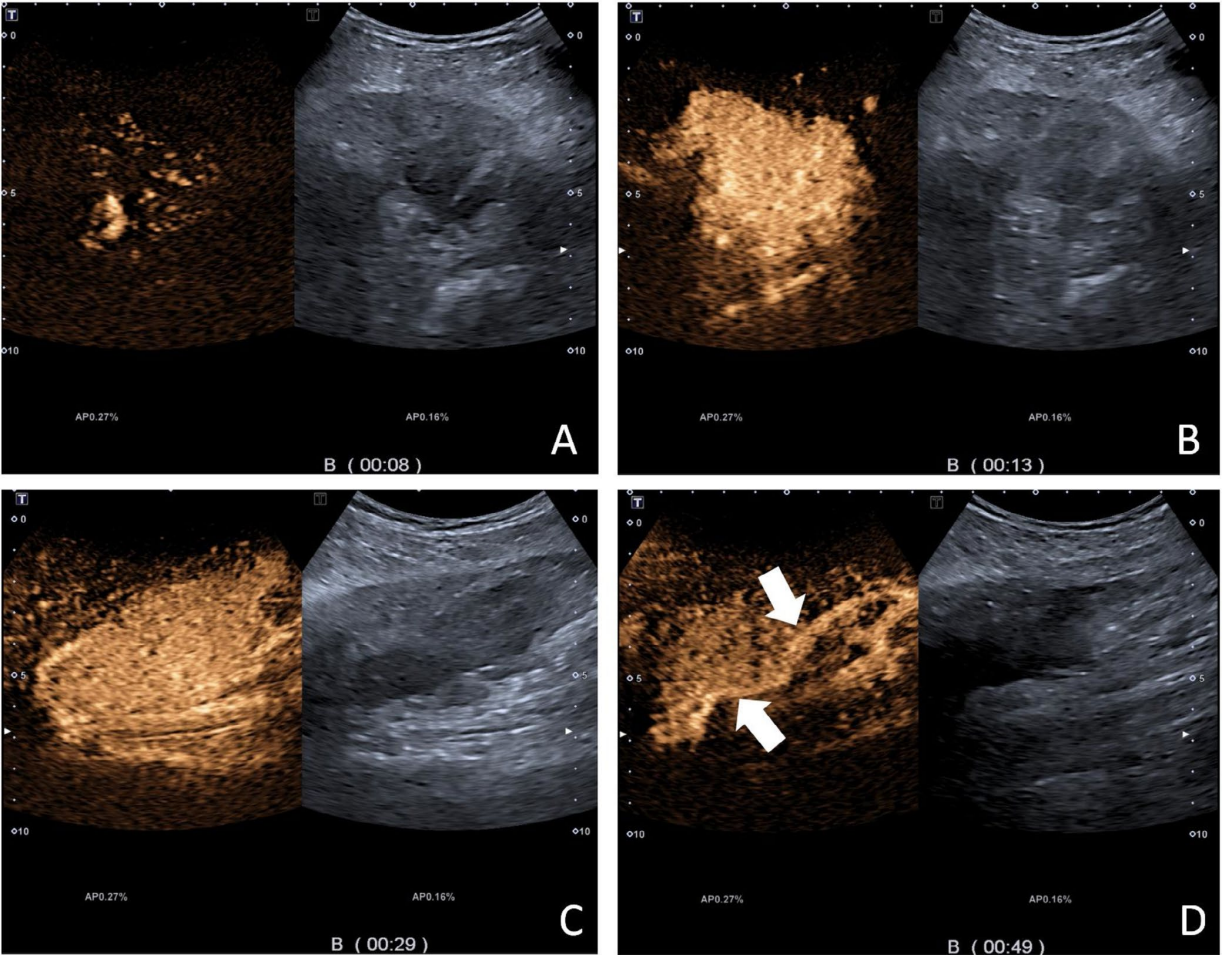
(A) CEUS shows the lesion begins enhancing 8 s before the surrounding muscle tissue. (B, C) The lesion shows stronger enhancement than the surrounding muscle tissue during the arterial phase, with unclear boundaries. (B) Transverse and (C) longitudinal views. (D) Large perforating vessels within the lesion (white arrow).

**FIGURE 3:**
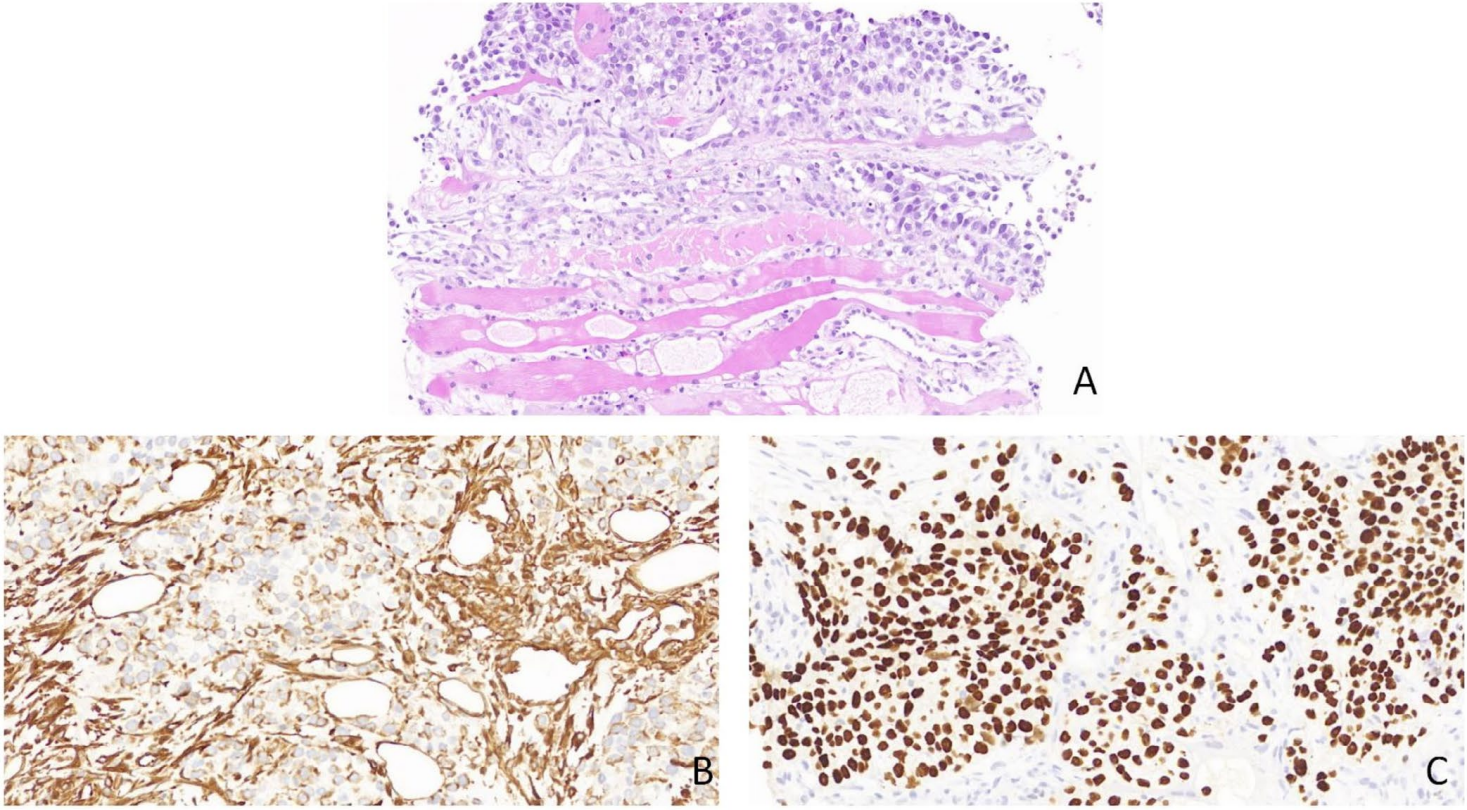
Histopathological confirmation of the muscle lesion. (A) Hematoxylin and eosin (H&E) staining (20× magnification). (B) and (C) Immunohistochemical staining (20× magnification). Histopathological examination of the biopsy specimen confirmed the presence of tumor tissue within the muscle, which was diagnostic of squamous cell carcinoma. Immunohistochemical results: CK5/6 (partial +), Ki‐67 (60% +), Vimentin (partial +), P63 (+), P40 (+), P53 (+), CK (+), TTF‐1 (−), Napsin A (−), S‐100 (−), Caldesmon (−), Desmin (−), Myoglobin (−).

A 58‐year‐old male patient underwent left lower lung resection for lung cancer 2 years ago. The postoperative staging was IA (T1aN0M0), and the pathological diagnosis was lung squamous cell carcinoma, with a lesion size of approximately 3 × 2 × 2 cm, infiltrating the surrounding lung tissue but without pleural involvement. Lymph nodes submitted for examination showed no metastasis. Pleural metastasis was diagnosed more than 9 months post‑surgery. After 17 months, a PET‐CT scan revealed metastasis to the supraclavicular lymph nodes and right parietal lobe. He underwent gamma knife radiosurgery for the lesions and was given chemotherapy. Two years post‐surgery, during inpatient chemotherapy, the patient developed bilateral calf swelling, and the clinicians suspected deep vein thrombosis in the calves. Lower limb venous ultrasound showed right‐sided peroneal vein thrombosis. In the left calf, an elongated hypoechoic area approximately 9.2 × 2.5 cm was found within the soleus muscle, with uneven internal echogenicity, unclear boundaries, and irregular shape. Color Doppler Flow Imaging (CDFI) showed blood flow signals within the lesion, and superb microvascular imaging (SMI) indicated blood flow signals (Figure 1). The presence of blood flow signals was inconsistent with a diagnosis of thrombosis, and it was difficult to distinguish between a muscle hematoma and a tumor. The patient then underwent CEUS, with 2.4 mL of Sonovue contrast agent injected via the antecubital vein. The left calf lesion began enhancing at 8 s, significantly earlier than the surrounding normal muscle tissue. Rapid and uneven enhancement was observed, with large feeding arteries and their branches seen around and within the lesion. The enhanced area was clearly larger than the gray‐scale ultrasound image, and the lesion's boundary was unclear, with slower enhancement compared to surrounding muscle tissue in the venous phase (Figure 2). Based on the CEUS findings and the patient's history, we suspected the solid, hypervascular lesion in the left calf soleus muscle to be a malignant tumor, with metastatic cancer being a possibility. An ultrasound‑guided biopsy was subsequently performed (Figure 3), and pathology confirmed squamous cell carcinoma.

## 3 Treatment and Follow‐Up

**FIGURE 4:**
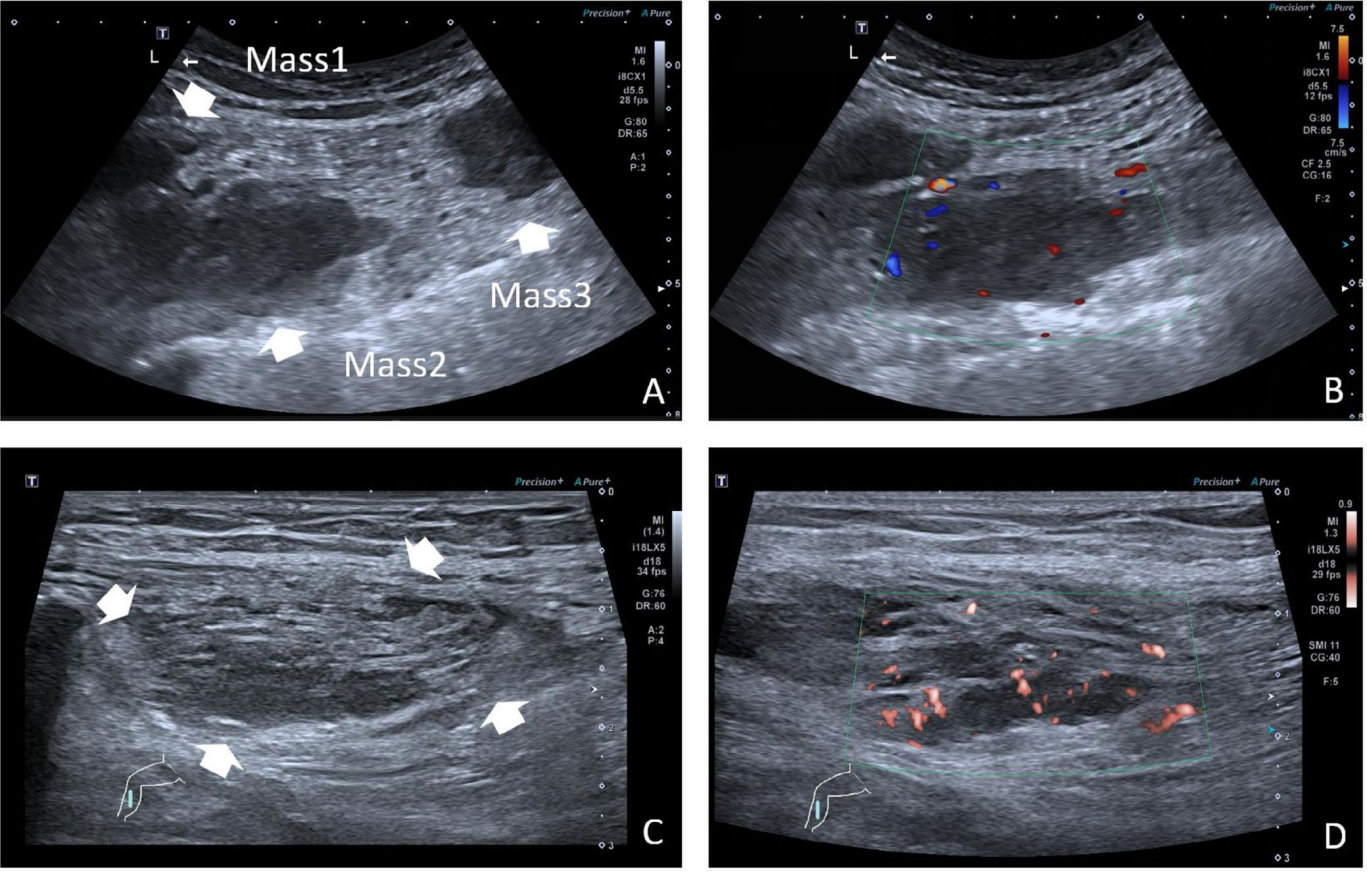
(A) Ultrasonographic image of multiple metastatic lesions in the left thigh. (B) CDFI image of metastatic lesions in the left thigh. (C) Ultrasonographic image of metastatic lesions in the right calf. (D) SMI of metastatic lesions in the right calf.

The patient subsequently underwent antitumor therapy with docetaxel combined with nedaplatin and anticoagulation therapy with rivaroxaban. After 10 days, the lower limb edema showed no significant improvement. A repeat lower extremity venous ultrasound revealed multiple patchy hypoechoic areas with indistinct borders and irregular shapes within the muscle layers of both thighs and calves (Figure 4). Based on the patient's medical history, these findings were considered indicative of metastatic tumors.

## 4 Discussion and Conclusion

Although muscle tissue occupies a large area of the body and has an abundant blood supply, SMM from LC is relatively rare. This is closely related to factors such as changes in local blood supply caused by muscle contraction and the acidic environment generated by lactate production [2]. SMM from LC most commonly occurs in the upper limbs and trunk, with a lower incidence in the lower limbs [3]. Depending on the location of the metastasis, patients may present with different clinical manifestations, such as being asymptomatic, having painless subcutaneous nodules, or experiencing local pain and swelling. The specificity of these clinical symptoms is relatively low, particularly when the lesions occur in the lower limbs, as these limbs are prone to swelling or traumatic changes. As a result, the clinical manifestations of SMM are more likely to be overlooked by clinicians. Table 1 summarizes the clinical manifestations and imaging diagnostic methods of metastatic lung cancer to the lower limbs as reported in the literature [3, 4, 5, 6, 7, 8]. The most commonly used imaging examinations for patients with muscle metastasis include radiological tests such as CT, MRI [2]. Previously, ultrasound was less frequently used to detect intramuscular metastases, serving primarily when patients presented with a palpable mass or for initial detection of an occult tumor. However, with the development and widespread use of ultrasound technology, particularly musculoskeletal ultrasound, ultrasound has become increasingly applied to detect lesions in muscle tissue. Additionally, due to its convenience, cost‐effectiveness, and lack of radiation, ultrasound plays an important role in the routine follow‐up of cancer patients. In this case, the SMM in the calf soleus muscle was discovered during a lower limb deep vein ultrasound examination conducted during hospitalization. Therefore, accurately understanding the appearance of muscle metastases in ultrasound and CEUS, as well as distinguishing them from other diseases, holds significant clinical importance.

**TABLE 1:** Clinical manifestations and imaging diagnostic methods of lower limb muscle metastases from lung cancer: A literature review.

Author	Journal	Year	Location	Age	Sex	Pathological types	Clinical symptoms	Imaging technology
Lin et al.	Translational Cancer Research	2019	Left thigh	64	Male	SCC	Pain, Touchable mass	US, MRI, CT
Aniqa et al.	Cureus	2024	Right thigh	60	Female	LUAD	Asymptomatic	CT, PET‐CT
Linda et al.	Cureus	2024	Right deltoid	54	Male	NSCLC	Pain	CT
Yuni et al.	American Journal of Case Reports	2024	Right thigh	47	Female	LUAD	Pain, Swelling	MRI, CT, PET‐CT
Sung et al.	Thoracic Cancer	2014	Left thigh	71	Male	SCC	Asymptomatic	PET‐CT
Hironari et al.	BMJ case report	2021	Right calf	77	Female	LUAD	Pain, Elastic hard mass	PET‐CT, MRI

Abbreviations: LUAD, lung adenocarcinoma; NSCLC, non‐small cell lung cancer; SCC, squamous cell carcinoma.

The ultrasound appearance of muscle metastases varies depending on the characteristics of the primary disease. According to literature, most metastases appear as heterogeneous hypoechoic lesions (98.6%) with clear borders (85.9%). The shape of the lesions is predominantly round or oval (47.9%), followed by lobulated and irregular shapes (42.2%). The blood flow signal within the lesions is relatively rich (64.8%) [9]. Notably, while intramuscular thrombi typically appear avascular on ultrasound, the neovascularization process during thrombus organization may yield detectable flow signals. In these diagnostically challenging scenarios, CEUS provides distinct advantages by clearly differentiating between the enhancement patterns of organizing thrombi and metastatic lesions. There are few reports in the literature regarding the CEUS appearance of skeletal muscle metastases, and directly determining the type of the primary disease via ultrasound is challenging. However, early identification of space‐occupying lesions in the muscle and performing biopsy on suspicious malignant lesions are still significant for the patient's prognosis. On CEUS, if the enhancement extends beyond the B‐mode borders, the boundaries become unclear, and the arrival time of the contrast agent in the lesion exceeds 9.5 s, the possibility of malignancy should be considered [10].

CEUS plays a crucial role in differentiating skeletal muscle metastases from benign lesions such as intramuscular or intermuscular hematomas, intramuscular abscesses, and myositis ossificans by demonstrating distinct microvascular perfusion patterns. Acute hematomas show a complete absence of enhancement, while chronic hematomas exhibit patchy enhancement due to neovascularization, abscesses display characteristic peripheral rim enhancement surrounding central necrosis, and myositis ossificans presents with central non‐enhancement and peripheral eggshell‐like enhancement, enabling reliable discrimination from the disorganized vascular patterns typical of metastatic lesions [11].

In diagnosing lower extremity muscle metastases in this case, while multimodal ultrasound provided valuable diagnostic assistance and successfully guided needle biopsy, certain limitations were evident. Current evidence demonstrates that combining multiple imaging modalities significantly improves diagnostic accuracy. Specifically, MRI offers superior soft tissue resolution, enabling more sensitive detection of intramuscular microhemorrhages and tumor infiltration, whereas ultrasound, despite its portability, remains operatordependent and limited by tissue characteristics. In this case, the similarity of symptoms to deep vein thrombosis precluded comprehensive multimodal evaluation. These findings suggest an optimized diagnostic pathway for similar cases: initial ultrasound screening followed by MRI for definitive characterization, with selective biopsy as needed. This approach would minimize invasive procedures while enhancing diagnostic precision, aligning with the principle of multimodality emphasized in virtopsy research [12].

## 5 Conclusion

This case underscores the diagnostic dilemma of SMM in LC, particularly when its clinical presentation closely mimics DVT. CEUS emerges as a crucial diagnostic tool by demonstrating pathognomonic enhancement patterns, including rapid heterogeneous hyperenhancement, lesion expansion, ill‐defined margins, and prominent penetrating vessels, which collectively facilitate reliable differentiation between metastatic lesions and thrombi. Clinicians should maintain a high index of suspicion for SMM in cancer patients presenting with unexplained limb swelling, particularly when unresponsive to anticoagulation therapy. Multicenter collaborative studies are imperative to establish evidence‐based, standardized CEUS diagnostic criteria for muscular metastases.

## Author Contributions

**Yiqun Lin:** conceptualization, data curation, investigation, methodology, writing – original draft, writing – review and editing. **Shuai Zheng:** writing – review and editing. **Hongxia Zhang:** writing – review and editing. **Sen Wang:** writing – review and editing. **Wen He:** writing – review and editing. **Wei Zhang:** writing – review and editing.

## Ethics Statement

The authors have nothing to report.

## Consent

Consent was given by the patient before writing the case report. Written informed consent was obtained from the patient for publication of this case report and any accompanying images. A copy of the written consent is available for review by the Editor‐in‐Chief of this journal.

## Conflicts of Interest

The authors declare no conflicts of interest.

## Data Availability

The data that support the findings of this study are available on request from the corresponding author. The data are not publicly available due to privacy or ethical restrictions.
